# Interaction of HDAC2 with SARS-CoV-2 NSP5 and IRF3 Is Not Required for NSP5-Mediated Inhibition of Type I Interferon Signaling Pathway

**DOI:** 10.1128/spectrum.02322-22

**Published:** 2022-09-29

**Authors:** Nenavath Gopal Naik, See-Chi Lee, Beatriz H. S. Veronese, Zhe Ma, Zsolt Toth

**Affiliations:** a Department of Oral Biology, University of Florida College of Dentistry, Gainesville, Florida, USA; b UF Genetics Institute, Gainesville, Florida, USA; c UF Health Cancer Center, Gainesville, Florida, USA; d Department of Molecular Genetics and Microbiology, University of Florida College of Medicine, Gainesville, Florida, USA; Oklahoma State University, College of Veterinary Medicine

**Keywords:** HDAC2, NSP5, SARS-CoV-2, type I IFN signaling, interferons, protein-protein interactions

## Abstract

Over the last 2 years, several global virus-host interactome studies have been published with SARS-CoV-2 proteins with the purpose of better understanding how specific viral proteins can subvert or utilize different cellular processes to promote viral infection and pathogenesis. However, most of the virus–host protein interactions have not yet been confirmed experimentally, and their biological significance is largely unknown. The goal of this study was to verify the interaction of NSP5, the main protease of SARS-CoV-2, with the host epigenetic factor histone deacetylase 2 (HDAC2) and test if HDAC2 is required for NSP5-mediated inhibition of the type I interferon signaling pathway. Our results show that NSP5 can significantly reduce the expression of a subset of immune response genes such as IL-6, IL-1β, and IFNβ, which requires NSP5’s protease activity. We also found that NSP5 can inhibit Sendai virus-, RNA sensor-, and DNA sensor-mediated induction of IFNβ promoter, block the IFN response pathway, and reduce the expression of IFN-stimulated genes. We also provide evidence for HDAC2 interacting with IRF3, and NSP5 can abrogate their interaction by binding to both IRF3 and HDAC2. In addition, we found that HDAC2 plays an inhibitory role in the regulation of IFNβ and IFN-induced promoters, but our results indicate that HDAC2 is not involved in NSP5-mediated inhibition of IFNβ gene expression. Taken together, our data show that NSP5 interacts with HDAC2 but NSP5 inhibits the IFNβ gene expression and interferon-signaling pathway in an HDAC2-independent manner.

**IMPORTANCE** SARS-CoV-2 has developed multiple strategies to antagonize the host antiviral response, such as blocking the IFN signaling pathway, which favors the replication and spreading of the virus. A recent SARS-CoV-2 protein interaction mapping revealed that the main viral protease NSP5 interacts with the host epigenetic factor HDAC2, but the interaction was not confirmed experimentally and its biological importance remains unclear. Here, we not only verified the interaction of HDAC2 with NSP5, but we also found that HDAC2 also binds to IRF3, and NSP5 can disrupt the IRF3-HDAC2 complex. Furthermore, our results show that NSP5 can efficiently repress the IFN signaling pathway regardless of whether viral infections, RNA, or DNA sensors activated it. However, our data indicate that HDAC2 is not involved in NSP5-mediated inhibition of IFNβ promoter induction and IFNβ gene expression.

## INTRODUCTION

Severe acute respiratory syndrome coronavirus 2 (SARS-CoV-2) is an enveloped positive-sense single-stranded RNA virus that belongs to the genus betacoronavirus within the Coronaviridae family (1, 2). SARS-CoV-2 is likely originated from bats and is responsible for the coronavirus disease 2019 (COVID-19) pandemic (3, 4). The SARS-CoV-2 genome encodes 16 nonstructural proteins (NSP1-16), 4 structural proteins, and at least 7 accessory proteins. While the nonstructural proteins make up the replication machinery and the structural proteins form the virion, the accessory proteins modulate the antiviral host response, although other viral proteins can also be involved (5, 6). Despite the tremendous research efforts since the beginning of the pandemic, the pathogenesis of SARS-CoV-2 remains largely unclear. We still need a better understanding of interactions between viral and host proteins that subvert antiviral host defenses and promote viral replication.

One of the first lines of host defense against viral infections is the activation of the type I IFN (IFN-I) signaling pathway by sensing viral nucleic acids in infected cells. Viral dsRNAs generated during replication and viral transcription can be detected by an array of nucleic acid receptors in the cytoplasm, such as the retinoic acid-inducible gene I (RIG-I), melanoma differentiation gene 5 (MDA5), or specific Toll-like receptors (TLRs), while the cGAS-STING pathway can serve as one of the several viral DNA sensors in the cytoplasm (7 to 9). Upon ligand binding, the nucleic acid receptors activate downstream signaling pathways that lead to the induction of innate immune responses by producing inflammatory cytokines, IFN-I, and other antiviral mediators. IFN-I acts in a paracrine fashion on neighboring cells by binding to the IFN-α/β receptor (IFNAR), which induces the expression of antiviral interferon-stimulated genes (ISGs) via the JAK/STAT signaling pathway (10).

The increased mortality of many severely ill COVID-19 patients has been linked to the excess production of proinflammatory cytokines (e.g., IL-6, IL-1β, TNF-α, and interferon) upon SARS-CoV-2 infection, causing multiple organ failure (11). Intriguingly, SARS-CoV-2 has also evolved multiple mechanisms to be able to suppress these antiviral immune responses in infected cells, which facilitates viral replication (12). Numerous studies have shown that several SARS-CoV-2 proteins can antagonize IFN-I production via distinct mechanisms. For example, while NSP6 binds to TANK-binding kinase 1 (TBK1) to suppress IRF3 phosphorylation, NSP13 inhibits TBK1 phosphorylation, and ORF6 protein interacts with karyopherin subunit alpha 2 (KPNA2) to inhibit the nuclear transport of IRF3 (13). Another report showed a dual function of the main protease NSP5 of SARS-CoV-2 that NSP5 can block both virus-triggered IFN-I production and the downstream IFN-I-mediated ISG induction (14).

A recent protein interaction screen revealed that NSP5 interacts with histone deacetylase 2 (HDAC2), but the interaction was not confirmed experimentally and the biological significance of this interaction has not been investigated (15). Since HDAC2 has been reported to play a positive regulatory role in IFNβ production and the expression of ISGs, we aimed to test if NSP5 could also inhibit the IFN-I signaling pathway by blocking HDAC2 activity (16, 17). Alternatively, it is also possible that NSP5 hijacks HDAC2 to inhibit the IFN-I signaling pathway. We found that NSP5 can inhibit the expression of proinflammatory cytokines such as IL-6, IL-1β, and IFNβ, which requires the protease activity of NSP5. We also identified specific ISGs whose expression was significantly reduced by NSP5. Our data also show that HDAC2 interacts with both NSP5 and IRF3, and NSP5 can disrupt the interaction between HDAC2 and IRF3. However, our results suggest that HDAC2 is not involved in the NSP5-mediated inhibition of IFNβ expression.

## RESULTS

### Nuclear localization of SARS-CoV-2 NSP5 and the inhibitory effect of NSP5 on the expression of cytokines.

**FIG 1:**
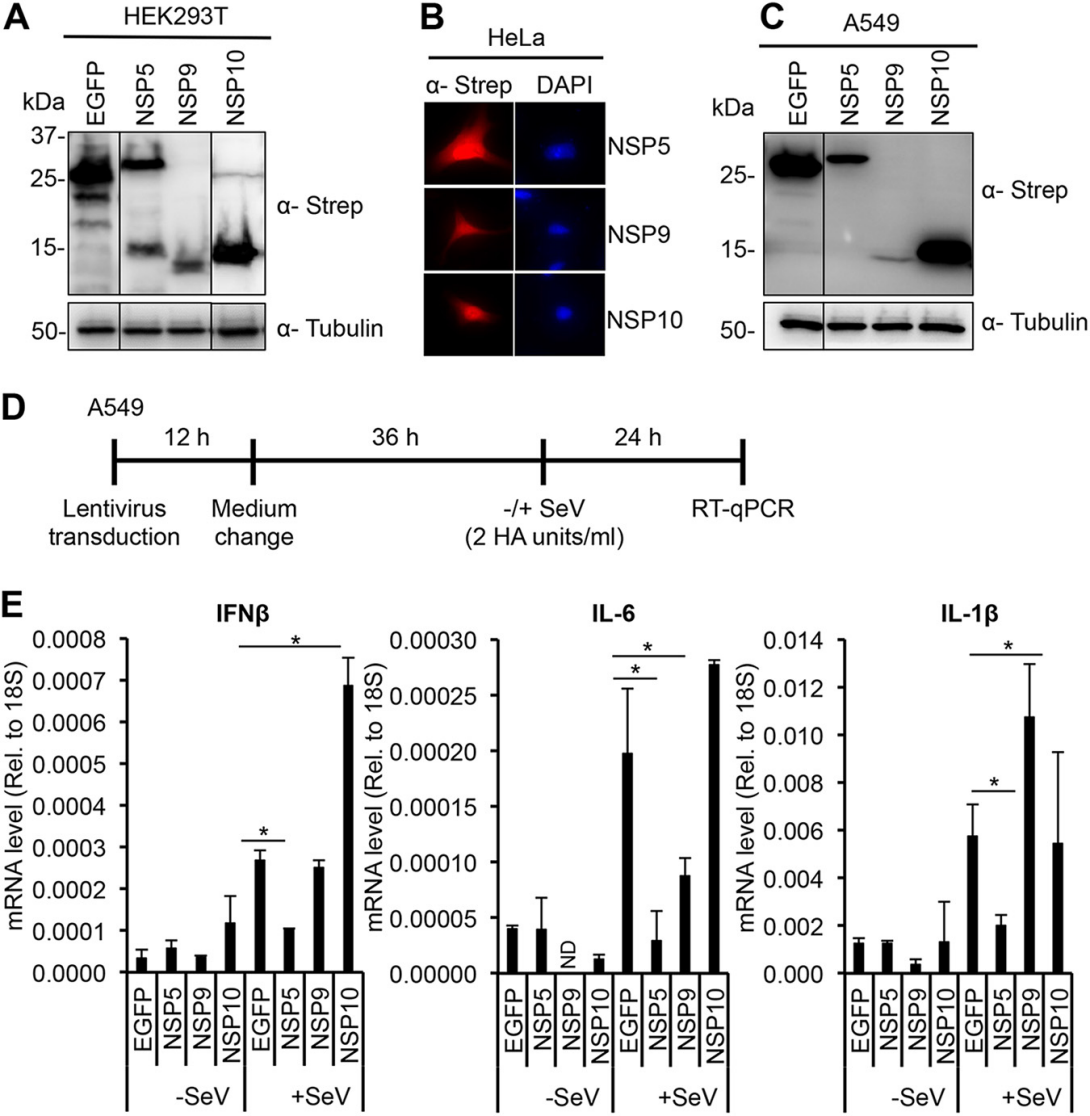
Expression, subcellular localization, and the effect of SARS-CoV-2 nuclear proteins on SeV-induced cytokines in epithelial cells. (A) Immunoblot analysis of the expression of 2×-Strep tagged NSP5, NSP9, NSP10, and EGFP in transfected HEK293T cells. (B) IFA was performed on transfected HeLa cells using an anti-Strep antibody. Nucleus is indicated by DAPI staining. (C) Expression of the 2×-Strep tagged viral proteins and EGFP in A549 cells using lentiviral transduction. (D) Experimental flowchart. After 48 h postransduction, A549 cells were treated with SeV (2 HA units/mL) for 24 h. (E) Cytokine gene expressions are measured by RT-qPCR. *t* tests were performed compared to EGFP in +SeV samples, and *P* of <0.05 (*) was considered statistically significant.

A recent study that determined the subcellular localization of FLAG-tagged SARS-CoV-2 proteins in transfected HEp-2 and Caco-2 epithelial cell lines revealed that several SARS-CoV-2 proteins such as NSP1, NSP5, NSP9, NSP10, and NSP13 can also be localized in the nucleus (18). We confirmed that the C-terminally 2×Strep epitope-tagged NSP5, NSP9, and NSP10, which showed similar expression levels in transfected HEK293T cells (Fig. 1A), have both nuclear and cytoplasmic localizations in HeLa cells as well (Fig. 1B). Since the expression levels of NSP1 and NSP13 were below the detection limit, we did not investigate them further. Interestingly, it has been shown in several cases that RNA virus proteins displaying nuclear localization can regulate the expression of immune response genes (19, 20). Thus, we tested the effect of NSP5, NSP9, and NSP10 on the expression of IFNβ, IL-6, and IL-1β, which are some of the most frequently deregulated cytokines in COVID-19 patients. The viral proteins were first expressed in A549 lung epithelial cells using lentiviral transduction followed by Sendai virus (SeV) infection for 24 h (Fig. 1C and D). SeV was used to induce the expression of the tested cytokines. RT-qPCR analysis showed that NSP5 significantly reduced the mRNA expression levels of IFNβ, IL-6, and IL-1β, while NSP9 and NSP10 had distinct effects (Fig. 1E). NSP10 overexpression increased IFNβ gene expression but had no effect on the expression of IL-6 and IL-1β. In contrast, NSP9 significantly increased SeV-induced IL-1β, while reduced IL-6 expression but had no effect on IFNβ mRNA expression. Taken together, these data show that NSP5 can be localized in the nucleus and can strongly inhibit the expression of cytokine genes.

### The protease activity of NSP5 is required for the inhibition of IFNβ, IL-6, and IL-1β expression.

**FIG 2:**
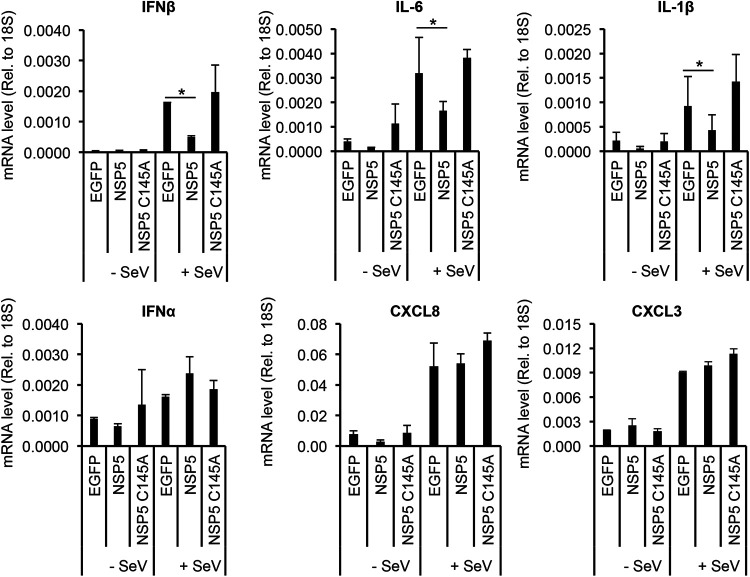
Protease activity of NSP5 is required for repressing the induction of cytokine genes. A549 cells were transduced with lentiviruses expressing the indicated SARS-CoV-2 proteins or EGFP as a negative control. At 48 h postransduction, the cells were treated with SeV (2 HA units/mL) for 24 h. Total RNA was extracted, and the expression of cytokine genes was analyzed by RT-qPCR. *t* tests were performed compared to EGFP in +SeV samples, and *P* of <0.05 (*) was considered statistically significant.

Since NSP5 functions as a viral protease of SARS-CoV-2 (21), we investigated whether its protease activity is needed for the inhibition of SeV-induced cytokine expression (Fig. 2). Furthermore, we wanted to determine if NSP5 has any specificity in inhibiting the expression of cytokine genes (Fig. 2). To this end, we expressed wild type (WT) or the enzymatically inactive mutant of NSP5 (C145A) in A549 cells and then infected the cells with SeV (22). We found that while WT NSP5 inhibited SeV-induced IFNβ, IL-6, and IL-1β gene expression, it did not show any effects on the expression of IFNα, CXCL8, and CXCL3. Interestingly, the C145A mutant of NSP5 failed to block SeV-induced IFNβ, IL-6, and IL-1β gene expression. These results indicate that NSP5 requires its protease activity to inhibit the expression of specific cytokine genes.

### NSP5 abrogates RNA and DNA sensors-mediated IFNβ promoter induction.

**FIG 3:**
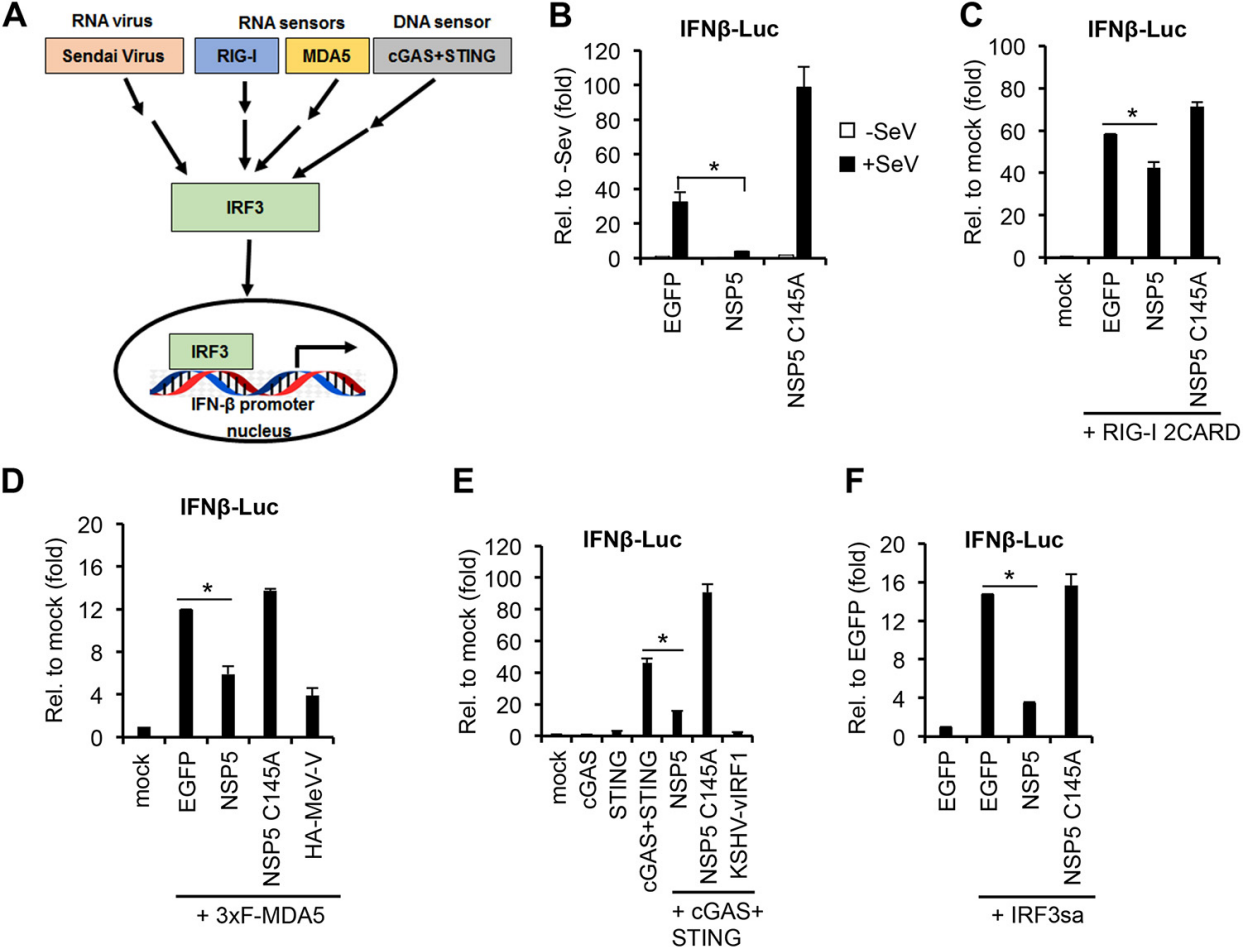
Testing the effect of NSP5 on IFNβ promoter induced by viral infection or different RNA or DNA sensors. (A) Schematic of the different modes of IFNβ promoter induction that were tested. Each tested pathway transmits the activation through the transcription factor IRF3, which is required for the induction of IFNβ promoter. (B to F) Luciferase assays using HEK293T cells that were cotransfected with an IFNβ promoter luciferase reporter plasmid (IFNβ-Luc) and plasmids expressing NSP5, NSP5 mutant C145A, or EGFP (negative control). The fold change was calculated by comparing luciferase activities to the basal activity of the IFNβ-Luc plasmid cotransfected with EGFP or IFNβ-Luc alone (mock). *t* tests were performed between NSP5 and EGFP samples, and *P* of <0.05 (*) was considered statistically significant. (B) IFNβ promoter was induced with SeV infection (2 HA units/mL) for 24 h. (C) IFNβ promoter was induced with the expression of the constitutively active RIG-I 2CARD. (D) IFNβ promoter was induced with the expression of 3×FLAG-MDA5. HA-MeV-V was used as a positive control for the inhibition. (E) IFNβ promoter was induced with cotransfection of cGAS and STING. KSHV vIRF1 was used as a positive control for the inhibition. (F) A constitutively active mutant of IRF3 (IRF3sa) was used for IFNβ promoter induction.

It was reported that SARS-CoV-2 infection does not induce robust IFN response in patients, indicating that the virus can efficiently block the activation of the IFN signaling pathway (23). Our data above are in line with previous results showing that NSP5 can strongly inhibit IFNβ expression (14, 24 to 27). Importantly, IFNβ expression can be induced through different IRF3-mediated signaling pathways, which can be activated by distinct nucleic-acid-sensing receptors and viral infections (Fig. 3A). Thus, we aimed to test whether NSP5 can inhibit IFN signaling pathways regardless of how they were activated. For this, we performed a series of IFNβ promoter luciferase reporter assays (Fig. 3B to F). First, HEK293T cells were cotransfected with the luciferase reporter plasmid and WT or C145A mutant NSP5. Afterward, the IFNβ signaling pathway was induced with SeV infection (Fig. 3B) or cotransfection of the RNA sensors RIG-I 2CARD (Fig. 3C) and 3×FLAG-MDA5 (Fig. 3D), the DNA sensor cGAS-STING (Fig. 3E), or IRF3sa, which is a constitutively active form of IRF3 (Fig. 3F) (28). We note that enhanced green fluorescent protein (EGFP) transfection was used as a negative control. In addition, the measles virus V protein (MeV-V) and the Kaposi’s sarcoma-associated herpesvirus (KSHV) protein vIRF1 were used as positive controls for inhibition of MDA5- and cGAS-STING-mediated IFN signaling pathways, respectively (29, 30). The experiments showed that WT NSP5 can inhibit the induction of IFNβ promoter regardless of how the IFN signaling pathway was activated. In contrast, the protease mutant C145A cannot repress the IFN signaling pathways supporting our data shown in Fig. 2 that the enzymatic activity of NSP5 is required for being able to block IFNβ promoter induction and thereby IFNβ gene expression.

### NSP5 inhibits IFNβ-induced signaling pathway.

**FIG 4:**
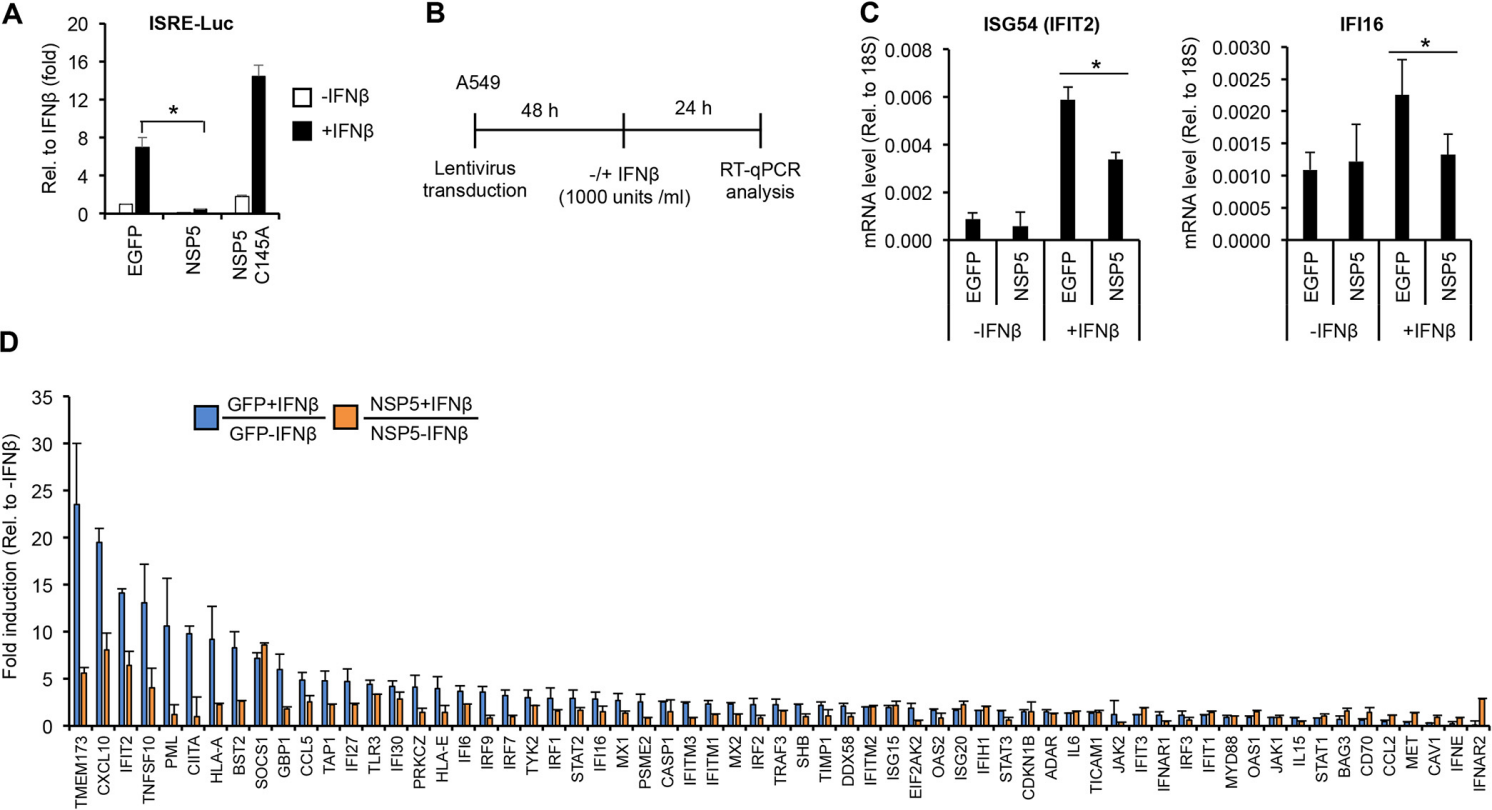
NSP5 inhibits the expression of interferon-stimulated genes. (A) Luciferase assay to test if NSP5 inhibits IFNβ-mediated activation of an interferon-stimulated response element (ISRE)-driven promoter (ISRE-Luc). HEK293T cells were cotransfected with the ISRE promoter luciferase reporter vector and the indicated expression plasmids. At 48 h posttransfection, the cells were treated with IFNβ (1000 IU/mL) for 24 h before harvesting them for luciferase assay. (B) Experimental flowchart. (C) RT-qPCR analysis of the expression of two ISGs in IFNβ-treated A549 cells expressing EGFP or NSP5. (D) The expression of type I IFN pathway-related genes was analyzed by RT-qPCR array. Error bars represent standard deviation (*n* = 2). *t* tests in panels A and C were performed between NSP5 and EGFP samples, and *P* of <0.05 (*) was considered statistically significant.

We further examined whether NSP5 also inhibits the downstream signaling pathway of the type I IFN receptor, which will lead to the reduction of interferon-stimulated gene (ISG) expression (Fig. 4). The expression of ISGs is regulated by the JAK-STAT pathway, which induces ISGs through gene promoters containing interferon-stimulated response element (ISRE) (31). Thus, we first performed an ISRE luciferase reporter assay to test NSP5 (Fig. 4A). HEK293T cells were transfected with an ISRE luciferase reporter plasmid (ISRE-Luc) along with an EGFP vector (negative control) or with a plasmid-expressing WT or C145A mutant NSP5, and then the cells were treated with IFNβ for 24 h. The results show that WT NSP5 but not its mutant C145A can inhibit IFNβ-induced ISRE promoter activity (Fig. 4A). Next, we tested if NSP5 can repress IFNβ-induced expression of ISGs in A549 cells (Fig. 4B to D). NSP5 was expressed in the IFNβ-treated A549 cells using lentivirus transduction. RT-qPCR analysis showed that NSP5 resulted in downregulation of ISG54 (also known as IFIT2) and IFI16 expression in A549 cells (Fig. 4C). Based on this observation, we extended the gene expression analysis for 84 human type I IFN response-related genes (Fig. 4D). We found that the expression of several ISGs was decreased in the NSP5-expressing cells compared to cells expressing GFP in the presence of IFNβ (Fig. 4D). Altogether, these results indicate that NSP5 can inhibit the type I IFN response pathway as well.

### NSP5 disrupts the interaction between HDAC2 and IRF3 in SeV-infected cells.

**FIG 5:**
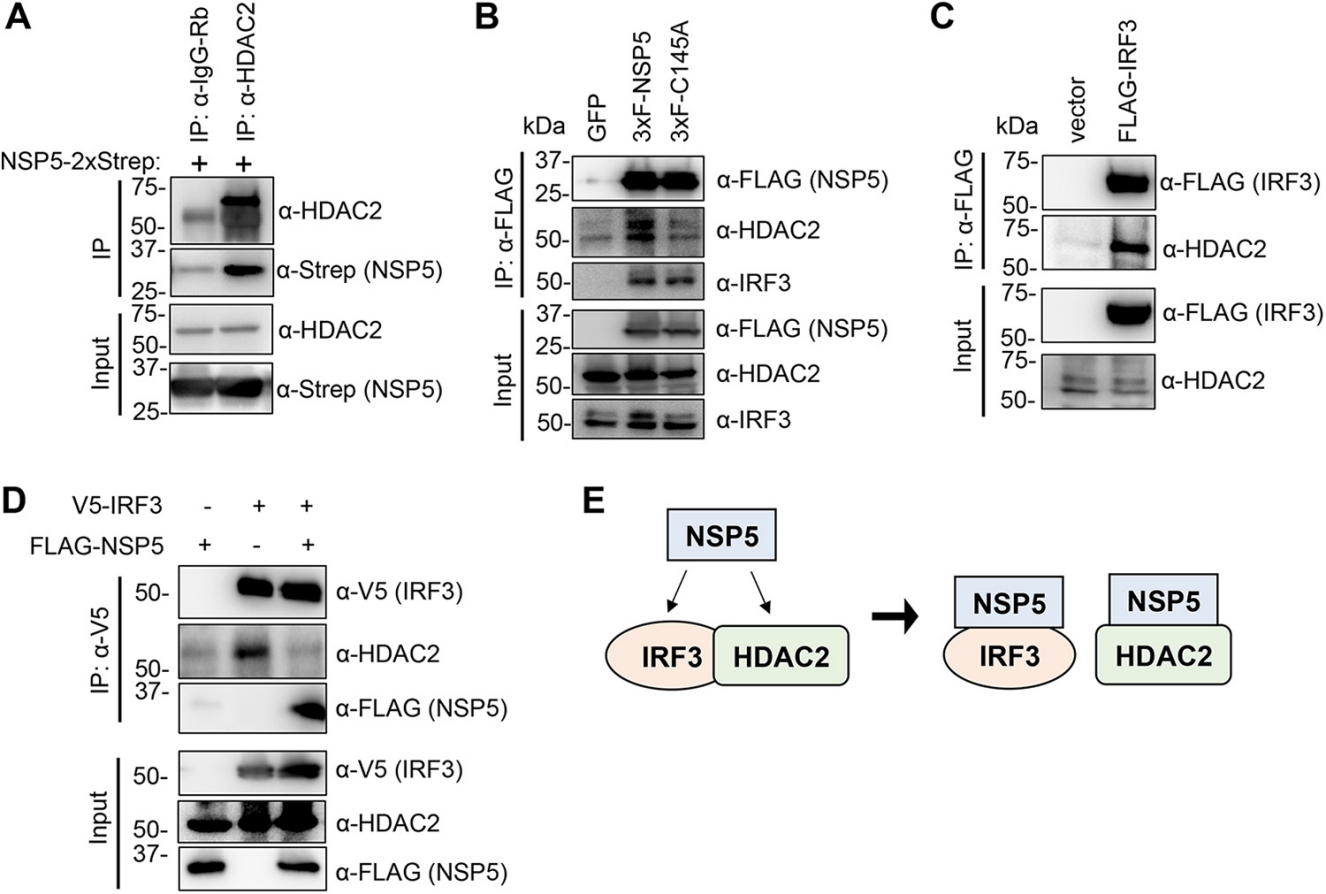
NSP5 interacts with HDAC2 and IRF3. (A) HEK293T cells were transfected with NSP5-2×Strep, and the immunoprecipitation (IP) was performed with HDAC2 or IgG (rabbit) antibodies. (B) FLAG IP was performed using HEK293T cells transfected with 3×FLAG-NSP5 or 3×FLAG-NSP5 mutant C145A. (C) HEK293T cells were transfected with FLAG-IRF3, and the IPs were carried out with anti-FLAG antibody. (D) HEK293T cells were transfected with V5-IRF3 and 3×FLAG-NSP5 for 48 h and then treated with SeV for 24 h. The IPs were performed with anti-V5 antibody. (E) Schematic of interactions between NSP5, IRF3, and HDAC2. We note that these interactions are not necessarily direct–direct protein interactions, which have yet to be shown.

A previous global virus–host protein interactome study indicated that the epigenetic factor histone deacetylase 2 (HDAC2) interacts with WT NSP5 but not its enzymatically dead mutant (C145A) (15). However, the interaction was not confirmed experimentally. To determine if there is an interaction, we performed an immunoprecipitation (IP) assay with anti-IgG (negative control) and anti-HDAC2 antibodies using HEK293T cells transfected with NSP5-2×Strep (Fig. 5A). We found that NSP5-2×Strep does interact with HDAC2 (Fig. 5A). We also carried out a FLAG IP using HEK293T cells expressing 3×FLAG-NSP5 or the 3×FLAG-NSP5 C145A mutant (Fig. 5B). We verified that WT NSP5 but not the C145A mutant can interact with HDAC2. In contrast, we found that both WT and the C145A mutant can be coimmunoprecipitated with IRF3 (Fig. 5B). In addition, our data show that HDAC2 can also bind to IRF3 but that this interaction is disrupted by NSP5 (Fig. 5C and D). Taken together, these results indicate that IRF3 and HDAC2 interact with each other but that NSP5 can abolish their complex by binding to both IRF3 and HDAC2 (Fig. 5E).

### HDAC2 can modulate the activation of interferon signaling pathway.

**FIG 6:**
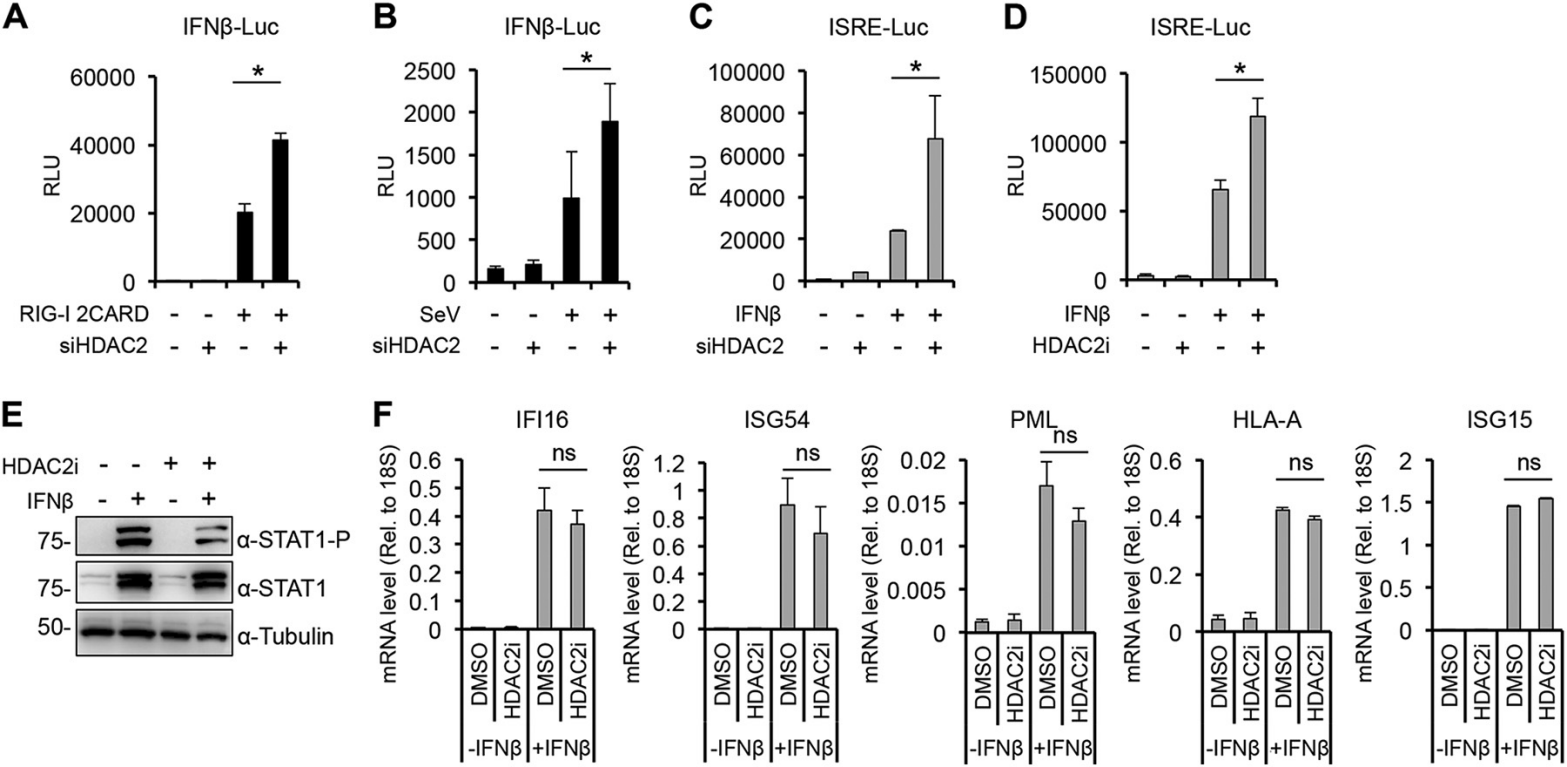
Testing the effect of HDAC2 on the activation of type I IFN signaling pathway. Luciferase assay with siControl- and siHDAC2-treated HEK293T cells that were transfected with IFNβ-Luc reporter plasmid, and the IFNβ promoter was induced with (A) cotransfection of RIG-I-2CARD or with (B) SeV infection (2 HA units/mL). (C and D) Luciferase assay with HEK293T cells that were transfected with ISRE-Luc reporter plasmid and also treated with HDAC2 siRNA (C) or HDAC2 inhibitor (D). The cells were induced with IFNβ (500 IU/mL) for 24 h before being harvested for the luciferase assay. (E and F) A549 cells treated with 10 μM HDAC2 inhibitor were induced with 500 IU/mL of IFNβ for 24 h. Subsequently, immunoblot analysis was performed (E), and RT-qPCR was used for analyzing the expression of ISGs (F). *t* tests were performed, and *P* of <0.05 (*) was considered statistically significant; ns, not significant.

The interaction between IRF3 and HDAC2 suggests that HDAC2 may play a role in the regulation of the IFN signaling pathway. To test this, we first performed an IFNβ promoter luciferase reporter assay in which the IFNβ promoter of the reporter plasmid was induced by cotransfecting RIG-I 2CARD or SeV infection while HDAC2 expression was inhibited by siRNA (Fig. 6A and B). We found that siHDAC2 slightly but significantly increased the induction of IFNβ promoter compared to the siControl samples. To examine the effect of HDAC2 on the type I IFN response pathway, siHDAC2- or HDAC2 inhibitor (HDAC2i)-treated HEK293T cells were transfected with ISRE reporter plasmid and then treated with IFNβ (Fig. 6C and D). The results show that both the depletion and the inhibition of HDAC2 slightly increased IFNβ-triggered ISRE promoter activation. These data imply that HDAC2 can act as a negative regulator of both IFNβ promoter and IFNβ-induced ISRE promoters. To verify the role of HDAC2 in the regulation of the type I interferon signaling pathway, we tested the effect of the HDAC2 inhibitor on STAT1 activation and the induction of ISGs (Fig. 6E and F). A549 cells were treated with HDACi or dimethyl sulfoxide (DMSO; negative control) and then stimulated with IFNβ. We found that although the phosphorylation of STAT1, which indicates its activation, was slightly reduced by HDAC2i, the expression of tested ISGs was not affected (Fig. 6E and F). These results indicate that HDAC2 can modulate the activation of IFNβ and ISRE promoters, but its effect is probably limited in the global regulation of the expression of ISGs.

### HDAC2 is not required for NSP5-mediated inhibition of type I interferon signaling pathway.

**FIG 7:**
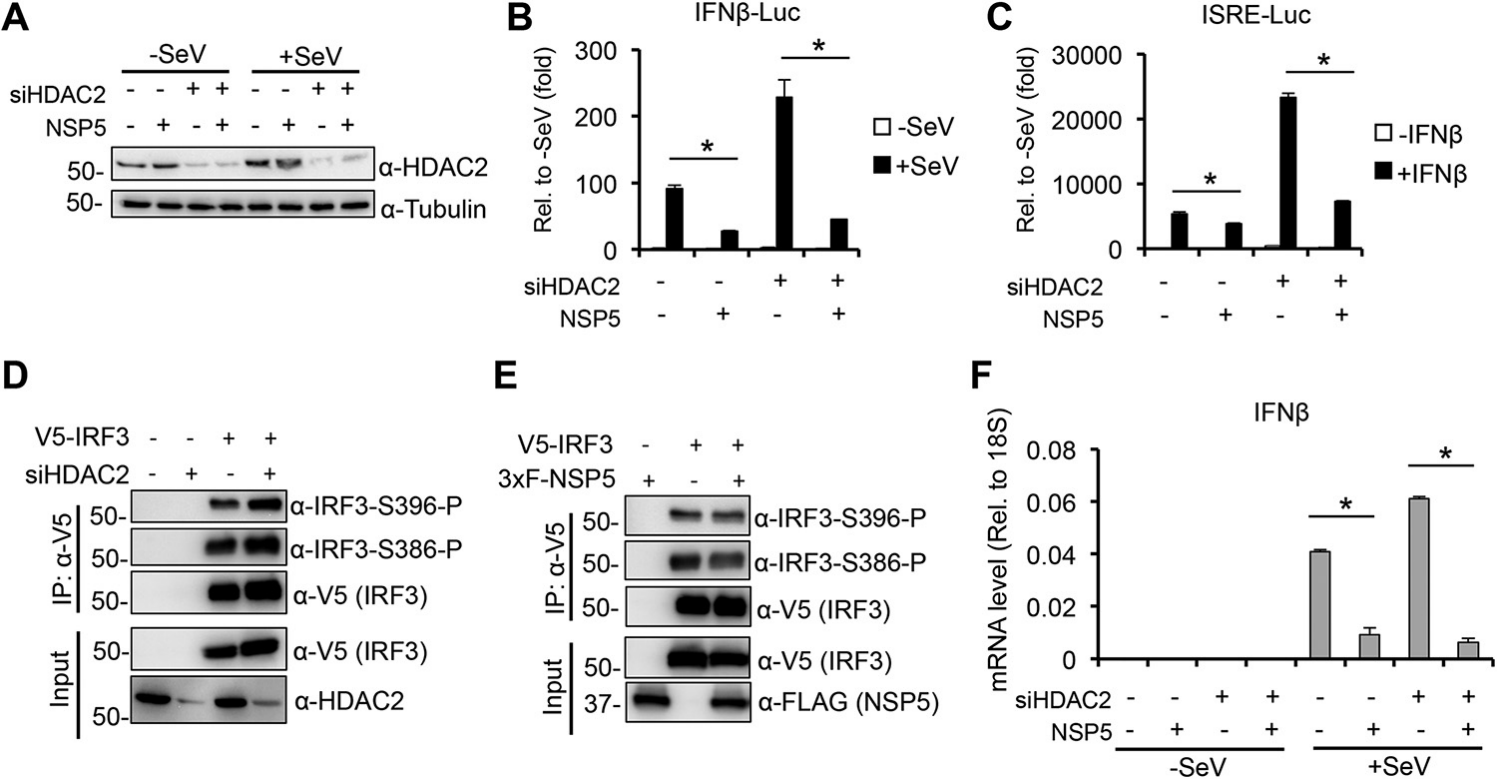
HDAC2 is dispensable for NSP5-mediated inhibition of the type I interferon signaling pathway. siControl- and siHDAC2-treated HEK293T cells were cotransfected with NSP5 and IFNβ-Luc followed by SeV infection, and then (A) immunoblot analysis and (B) luciferase assay were performed. (C) Luciferase assay with ISRE-Luc in siHDAC2-treated 293T cells. We used 500 IU/mL of IFNβ for induction. (D) siControl- and siHDAC2-treated HEK293T cells were transfected with V5-IRF3, and the IPs were performed with an anti-V5 antibody. (E) HEK293T cells were cotransfected with V5-IRF3 and 3×FLAG-NSP5. The IPs were performed with an anti-V5 antibody. (F) siControl- and siHDAC2-treated A549 cells were transduced with lentivirus-expressing EGFP (negative control) or NSP5 followed by SeV infection (2 HA units/mL) for 24 h. IFNβ gene expression was determined by RT-qPCR.

Based on our finding that NSP5 can disrupt the interaction between HDAC2 and IRF3 (Fig. 5), we wanted to examine if NSP5 utilizes HDAC2 for blocking the induction of IFNβ promoter and the activation of the IFN response signaling pathway (Fig. 7). To test this, we used siRNA to deplete HDAC2 in HEK293T cells and then transfected the cells with NSP5, and IFNβ promoter or ISRE promoter luciferase reporter plasmids (Fig. 7A to C). The transfected cells were either infected with SeV or stimulated with IFNβ to induce the IFNβ promoter (Fig. 7B) or ISRE promoter (Fig. 7C), respectively. Figure 7A shows the efficiency of siRNA inhibition of HDAC2. We found that while siHDAC2 results in a 2- to 3-fold increase of IFNβ and ISRE promoter activities as are also shown in Fig. 6, siHDAC2 did not affect NSP5-mediated repression of IFNβ and ISRE promoter activation (Fig. 7B and C). We also tested the effect of siHDAC2 and NSP5 on the phosphorylation of IRF3, which is required for the induction of IFNβ promoter. We found that neither siHDAC2 nor NSP5 overexpression affected IRF3 phosphorylation (Fig. 7D and E). Finally, we tested if HDAC2 is required for NSP5-mediated inhibition of endogenous IFNβ gene expression (Fig. 7F). We expressed NSP5 in siControl- and siHDAC2-treated A549 cells and then infected the cells with SeV to induce IFNβ gene expression. Our results show that NSP5 can still strongly downregulate IFNβ gene expression in siHDAC2-treated cells (Fig. 7F). Taken together, these results support the notion that HDAC2 is not required for NSP5-mediated IFNβ gene repression.

## DISCUSSION

A recent global protein interactome screen indicated that the main viral protease NSP5 of SARS-CoV-2 interacts with the transcription regulatory factor HDAC2 (15). However, the interaction was not confirmed experimentally, and its biological significance remains unknown. In this study, we verified that HDAC2 interacts with WT NSP5 but not with the protease mutant. In addition, the novel finding of our study is that we also showed an interaction between HDAC2 and IRF3, which can be disrupted by NSP5. We found that NSP5 can inhibit the expression of a subset of SeV-induced cytokines, which requires NSP5’s protease activity. While we could provide evidence for HDAC2 being a negative regulator of type I IFN signaling pathway, our results indicate that HDAC2 is not involved in NSP5-mediated inhibition of IFNβ gene expression and interferon signaling pathway. We acknowledge that the interferon-antagonizing properties of NSP5 may differ between SARS-CoV-2-infected cells versus NSP5-transfected cells. Therefore, further studies will be required to determine the role of HDAC2 in NSP5-regulated cellular processes in SARS-CoV-2-infected cells.

Although several virus–host protein interactome analyses have been performed with SARS-CoV-2 factors, none of them identified the components of the type I IFN signaling pathway as targets of NSP5 (15, 32 to 34). Also, there are two recent reports in which an unbiased screening of SARS-CoV-2 proteins was carried out to identify viral factors that can inhibit IFNβ production and type I IFN signaling pathway, but NSP5 was not found to affect these antiviral host responses (13, 35). However, our study supports the finding of several other previous studies that NSP5 can repress type I IFN signaling pathways (14, 24 to 27, 36). We speculate that the different expression levels of NSP5 and cell culture conditions may account for this discrepancy. Also, multiple distinct mechanisms have been identified for NSP5-mediated repression of the type I IFN signaling pathways by different research groups. It was shown that NSP5 can modulate the subcellular localization of IRF3 or antagonize the function of different components of the IFN response pathway such as RIG-I, MAVS, STING, and STAT1, resulting in the downregulation of both IFNβ production and the JAK-STAT signaling, which abrogates innate antiviral immunity (14, 24 to 26, 36). One of our novel findings is that we showed binding of NSP5 to IRF3 as well, and while the protease activity of NSP5 is not required for its binding to IRF3, it is necessary for NSP5-mediated inhibition of IFNβ promoter induced by IRF3 (Fig. 2, 3, and 5). These results are in line with previous studies supporting the notion that NSP5 of SARS-CoV-2 can efficiently inhibit IFN signaling pathway by targeting its different components at multiple levels, which requires the protease activity of NSP5 in some cases (24, 27). Importantly, NSP5 of SARS-CoV, porcine deltacoronavirus, and the alphacoronavirus porcine epidemic diarrhea virus have also been shown to block the IFN-I signaling pathway, indicating that NSP5-mediated inhibition of the antiviral IFN signaling pathway is evolutionarily conserved in the family Coronaviridae (27, 37, 38).

Another intriguing and novel finding of our study is that we showed an interaction between IRF3 and HDAC2, which can be abrogated by NSP5 (Fig. 5C and D). HDAC2 is a member of the lysine deacetylases family that can control the acetylation level of both histones and a variety of nonhistone proteins (39). HDACs can also regulate the innate antiviral response and IFNβ gene expression (40). It was shown in murine primary macrophages that HDAC1 and HDAC2 are important for phosphorylation and binding of IRF3 at the IFNβ promoter during gammaherpesvirus infection (16). Another study demonstrated that the lysine acetyltransferase KAT8 can acetylate IRF3 at lysine residue 359 in murine cells, which inhibits the binding of acetylated murine IRF3 to IFNβ promoter, resulting in reduced IFNβ production activity (41). We noticed that lysine 359 is conserved between mouse and human IRF3. Thus, we hypothesized that (i) HDAC2 may also function as a positive regulator of the type I IFN pathway in human cells by controlling the acetylation level and the activity of human IRF3, and (ii) NSP5 blocks IFNβ gene expression by interrupting HDAC2-IRF3 interaction and thereby increasing IRF3 acetylation. In addition, a recent study demonstrated that NSP5 of different coronaviruses, including SARS-CoV-2, can cleave HDAC2, which reduces the antiviral activity of HDAC2 (42). However, contrary to our expectation, we found that both siHDAC2 and an HDAC2 inhibitor increased the induction of both IFNβ and ISRE promoters and did not affect NSP5-mediated repression of the type I IFN signaling pathway. Thus, we concluded that HDAC2 plays an inhibitory role in type I IFN signaling pathway and that, although NSP5 can disrupt HDAC2-IRF3 interaction, HDAC2 is dispensable for NSP5-mediated repression of the IFN pathway. It is important to note that beyond the role of IRF3 in the induction of type I IFNs, IRF3 is also involved in the regulation of the expression of other cytokines and ISGs as well as in controlling apoptosis (43). Whether HDAC2 plays a role in any of these biological processes by interacting with IRF3 and NSP5 modulates them by interfering with the IRF3-HDAC2 complex formation awaits further studies.

## MATERIALS AND METHODS

### Cell lines and virus infection.

HEK293T (ATCC) and HeLa (NIH AIDS Reagent Program) cells were maintained in Dulbecco’s Modified Eagle Medium (DMEM) supplemented with 10% fetal bovine serum (FBS) and penicillin-streptomycin (P/S). Lung epithelial A549 (ATCC) cells were maintained in F-12K medium supplemented with 10% FBS and P/S. For experiments using Sendai virus (SeV), the cells were infected with 2 HA/mL of SeV Cantell strain (Charles River Laboratories). Lentivirus production and lentiviral transduction were performed as described previously (44).

### Antibodies, plasmids, and reagents.

The following antibodies were used in the study: anti-Strep Tag (Sigma, SAB2702216), anti-FLAG (Sigma, F1804), anti-V5 (Invitrogen, MA5-15253), anti-IRF3 (Cell Signaling, 4302S), anti-HDAC2 (Abcam, ab124974), anti-Tubulin (Sigma, T5326), anti-STAT1 (Cell Signaling, 14994), anti-STAT1-P (Cell Signaling, 9167), anti-IRF3-S386-P (Abcam, ab76493), and anti-IRF3-S396-P (Cell Signaling, 4947S). Human IFNβ was from Peprotech (300-02BC). The HDAC2 inhibitor Santacruzamat A was from Selleckchem.com (S7595). The HDAC2 siRNA was purchased from Santa Cruz Biotechnology (sc-29345). Lipofectamine RNAiMAX (Invitrogen) was used for the siRNA transfections, which were performed according to the manufacturer’s instructions. 3×FLAG-NSP5 was expressed from the pCDH-CMV-MCS-EF1-Puro vector. The expression plasmids for 2×Strep tagged NSP5, NSP9, NSP10, and EGFP were purchased from Addgene. FLAG-IRF3 and IFNβ-Luc plasmids were gifts from Katherine A. Fitzgerald (UMass Medical School) and Zhijian Chen (UT Southwestern), respectively. The ISRE-Luc reporter vector and the HA-MeV-V expression plasmid were provided by Takeshi Saito (University of Southern California) and Michaela Gack (Cleveland Clinic Florida Research & Innovation Center), respectively.

### Immunofluorescence analysis.

The immunofluorescence analysis (IFA) was performed with HeLa cells as described previously (45). The cells were first stained with primary mouse monoclonal anti-Strep Tag antibody followed by incubation with anti-mouse Alexa Fluor 568 antibody (Invitrogen) for 1 h at room temperature. The cells were washed three times with washing buffer (phosphate-buffered saline [PBS] with 0.2% Tween 20) and then stained with 4′,6-Diamidino-2-Phenylindole, Dihydrochloride (DAPI) to visualize the nuclei. For imaging, a Revolve fluorescence microscope (Echo Laboratories) was used.

### Luciferase reporter assay.

HEK293T cells in 24-well plates were transfected with the IFNβ or ISRE promoter luciferase reporter plasmids together with other plasmids, as indicated in the graphs. Transfection was carried out by polyethylenimine (PEI). At 48 h posttransfection, cells were collected in 200 μL of lysis buffer (Dulbecco's phosphate-buffered saline [DPBS] with 0.5% Triton X-100). Twenty microliters of cell lysates were mixed with 20 μL of ONE-Glo luciferase substrate (Promega), and the luciferase activity was measured by Promega GloMax-Multi Detection System. All luciferase assays were carried out three times in triplicate.

### Total RNA isolation, and RT-qPCR analysis.

Total RNA purification from cells and qPCR analysis were performed as described previously (44). The DNA sequences of primers used in qPCR are listed in Table 1. Relative gene expression was calculated by using the 2^−ΔCt^ method, where the expression of the 18S gene was used for normalization. For significance test, we used a two-tailed Student's *t* test where *P* of <0.05 was considered significant. For the RT-qPCR array, we used an RT^2^ Profiler PCR Array composed of 84 target genes (Qiagen, Human Type I Interferon Response, PAHS-016Z) following the manufacturer's recommended protocol. Relative gene expression was first normalized to GAPDH, which was included in the array, and then we calculated the gene expression changes using the 2^−ΔΔCt^ method by comparing +IFNβ to –IFNβ samples.

**TABLE 1:** Primer sequences

Gene	Forward (5′ to 3′)	Reverse (5′ to 3′)
IFNβ	CAGCAATTTTCAGTGTCAGAAGC	TCATCCTGTCCTTGAGGCAGT
IL-6	ATGTAACAAGAGTAACATGTGTGA	AGTGATGATTTTCACCAGGCAAGT
IL-1β	CCAACTGGTACATCAGCACCT	AGGAAGACACAAATTGCATGG
IFNα	GTGAGGAAATACTTCCAAAGAATCAC	TCTCATGATTTCTGCTCTGACAA
CXCL8	AGCTCTGTGTGAAGGTGCAGT	TAAATTTGGGGTGGAAAGGTT
CXCL3	GTCCGTGGTCACTGAACTGC	GGGGGACCTTACATTCACACT
ISG54	CTCAGAACGCCATTGACCCT	GGCTGCACTGCGAAGAACAT
IFI16	AGAAACAATGACCCCAAGAGC	CTTGGTGAAGAAACTGCTGGAT
18S	TTCGAACGTCTGCCCTATCAA	GATGTGGTAGCCGTTTCTCAGG

### Coimmunoprecipitation assay.

The transfected HEK293T cells were harvested at 48 h posttransfection for the immunoprecipitation (IP) experiments. The cells were washed once with cold PBS, lysed in NP-40 lysis buffer (50 mM Tris-HCl at pH 7.5, 150 mM NaCl, 0.5% NP-40, protease inhibitor cocktail from Roche), and passed through a 23-gauge needle 10 to 15 times, and then the cell lysates were incubated on ice for 15 min. After centrifugation, the cell lysates were subjected to preclearing using protein A-Sepharose 4B (ThermoFisher) for 2 h at 4°C and then incubated with antibodies overnight. The next day, protein A/G XPure agarose resin was added to the lysates, which were further incubated for 2 h at 4°C. The IPs were washed three times with the lysis buffer and then resuspended in 2× Laemmli buffer (Bio-Rad). The IP samples along with the input samples were analyzed by immunoblot.
